# Evolution of laughter from play

**DOI:** 10.1080/19420889.2024.2338073

**Published:** 2024-04-08

**Authors:** James A. Grant-Jacob

**Affiliations:** Faculty of Engineering and Physical Sciences, University of Southampton, Southampton, UK

**Keywords:** Deep learning, emotions, evolution, latent space, laughter, social behavior, tickling

## Abstract

In this hypothesis, I discuss how laughter from physical play could have evolved to being induced via visual or even verbal stimuli, and serves as a signal to highlight incongruity that could potentially pose a threat to survival. I suggest how laughter’s induction could have negated the need for physical contact in play, evolving from its use in tickling, to tickle-misses, and to taunting, and I discuss how the application of deep learning neural networks trained on images of spectra of a variety of laughter types from a variety of individuals or even species, could be used to determine such evolutionary pathways via the use of latent space exploration.

## 1. Introduction

Laughter is a vocal expression that can be both rhythmical and explosive [1] and is usually an involuntary act used to rapidly convey a signal of various valence in response to a stimulus [2], such as heavy tickling or slapstick comedy [3–5]. It can be accompanied with facial expressions such as smiling [6], can cause a sudden decrease in muscle strength known as cataplexy [7], and has been shown to release endogenous opioids in humans [8] and elevate pain threshold [9].

One prominent thesis regarding the function of laughter is incongruity theory, which claims that laughter is a response to something that violates our mental patterns and expectations [10–12]. In this hypothesis piece, I extend on such a theory by discussing how laughter could have evolved from physical play to visual or verbal stimuli in response to highlight incongruity that could be considered as having negative impact on survival, and it is an individual’s emotional state, social standing and situation that determines the usefulness of laughter, and thus, its valence. I discuss how deep learning neural networks could be applied to analyzing laughter to help test this hypothesis.

## 2. Origin in play

One way to potentially understand the function of laughter is to understand its origin. The most primitive form of laughter is arguably that triggered in play of non-human primates by the physical stimulus of heavy tickling [5,13,14], which occurs as a result of one individual trying to dominate another by temporarily incapacitating them though inducing muscle cataplexy [6]. Play in animals is often a way for individuals to learn survival techniques [3,15], and so since tickling generally occurs during play, and owing to the desirability not to be tickled and undergo cataplexy, avoiding a tickle could encourage an individual to develop defense and fighting abilities.

When a chimpanzee is tickled and produces the play pant (tickling induced laughter), it is communicating to the tickler and others around it that it is potentially temporarily incapacitated due to the induced cataplexy. But why would an individual highlight its own incongruity and that it is being dominated? The defensive mimic theory [16] suggests that human emotional expressions like laughter involve a complex set of facial expressions, vocalizations, and body postures, which evolved from defensive reflexes that protect the individual and act as a signal to regulate play by signaling a successful “attack” and in turn allows for reinforcing social bonds. Since laughter shows the traits of the smile [6], it could be considered a friendly expression and thus allow play to continue, as observed by [17], meaning that the individual is continued to be played with and learns to defend themselves.

For such a physically induced signal to display traits of happiness and smile, perhaps it evolved from another positive inducing interaction that occurs in response to another form of physical contact, such as grooming. Grooming releases endogenous opioids in both human and non-human primates [18,19], and such pleasure perhaps could have enabled the evolution of greater bonding and play. Indeed, Davilla-Ross and Palagi [20] discuss how expressions in animal play may communicate positive emotions to individuals of the same species and discuss how the motor resonance of these expressions increases bonding between the individuals. The benefits of positive emotions in play for bonding and the benefits of play itself could have therefore led to the potential for an individual to be incapacitated through too much arousal, as too much arousal can cause significant discomfort [21].

## 3. Evolution from play

At some point for some reason, if laughter originated from play interaction, laughter must have become disassociated from the requirement of physical contact. Since laughing in anticipation of tickling can be conditioned in human infants [22], the conditioning of laughing when being tickled perhaps allowed tickling induced laughter to occur without the need for physical contact. It has been demonstrated that laughter vocalizations can indeed occur as a result of anticipation of a tickle [23,24], allowing the potential for laughter to occur from tickle-related events in which individuals laugh when physical contact is minimal, such as in tickle failures that include avoiding a tickle (tickle-miss), escaping from a tickling attacker or other incongruent motion failures like slipping. Indeed, the incongruity theory of humor describes how humor-driven laughter highlights unexpected events [25,26], whilst superiority theory [27] claims individuals laugh at the misfortune of others to feel superior, and so the physical nature of tickle failures could be considered incongruent behavior, and the individual highlighting such incongruity by laughing, could also be seen as dominating. The sound of schadenfreude laughter and taunting laughter can be perceived as dominating [28], unlike tickling-induced laughter, which is perceived to have negative dominance. The highlighting of incongruent behavior is perhaps why fake laughter can be used to dominate another person and thus why fake laughter is perceived as dominant.

When we laugh at someone slipping on a banana peel, we are essentially communicating to them and others nearby that what they did was incongruent in a quick and loud action. Interestingly, high-ranking humans have been shown to produce more dominating sounding laughter than lower ranking individuals [29], so perhaps the advantage to an individual that is able to highlight physical failures by others is that they may more likely be seen as dominant. Although slipping and tripping are examples of incongruent behavior that can cause an individual to be laughed at, such events are potentially dangerous [30], and since vocal signals are used to improve survival chances of an individuals [31], laughter signals could therefore also help improve the fitness of a group as it is essentially teaching individuals in a group what is incongruent and how not to behave, which is possibly why the receiver of laughter can feel embarrassed [32]. Agreement in what individuals perceive as incongruent could be displayed in group laughter, as when comedians make comments or jokes, tell stories, and recall situations, it is thought that we are laughing in agreement at the situation they are reconstructing [33].

## 4. Testing hypothesis using deep learning

Understanding the origin and evolution of laughter, be it how tickling laughter evolved into different forms of laughter such as taunting, is arguably important in determining laughter’s function. One of the main methods of studying laughter is analyzing the acoustic spectra of different types of laughter in human and non-human primates. Winkler and Bryant [34] present a comparative review article of play vocalization and human laughter, drawing parallels between human laughter and animal play vocalizations and suggesting that laughter evolved from play-specific vocal signals in primates, which facilitate positive interactions and reduce the risk of aggression by acting as a regulation in social play. Their review proposes a basis for the analysis of play vocalizations across a variety of taxa to help understand the evolution of human social interaction, but state that appropriate acoustic signals found during play are sparse. Therefore, to be able to further understand laughter and play vocalization, and thus laughter’s evolution, it will be necessary to acquire a significant number of acoustic spectra. To do this using human input alone would be very time-consuming owing to the technical limitations in recording video footage and identifying the sound from such footage. As such, additional methods will need to be employed for data collection and analysis to aid in our understanding of laughter.

Deep learning convolutional neural networks [35], inspired by the primate visual cortex [36,37], have gained much interest in the past few years owing to their ability to classify, with a certain probability (confidence percentage), a vast number of objects better than humans [38] and do so with ever increasing performance [39,40]. These types of networks been used for understanding human cognition [41,42], whilst proving successful for posture detection [43], speech recognition [44], facial recognition [45], smile detection [46], and for identifying the songs from different birds [47,48]. In relation to laughter, deep learning convolutional neural networks have been used for laughter detection [49] and humor prediction [50–52] and could therefore be used to identify different types of laughter and vocalizations in humans and apes. Neural networks have already been used for video classification [53] and have already been demonstrated for chimpanzee facial recognition from videos in the wild [54]. Thus, such methodology could be extended to videos of laughter and play vocalizations to create a significantly large dataset, by using a neural network trained on a smaller human labeled dataset (i.e., human identified vocalizations and corresponding functions).

If sufficient data was acquired with appropriate functional labeling, to be able to use deep learning to help understand laughter’s evolution, one could train a convolutional neural network to correctly identify one type of laughter via their acoustic spectra and then apply the network to other types of laughter by the individual, in order to observe any similarities or trends. For example, if a deep learning convolutional neural network trained on determining tickle laughter is then applied to other tickle-related sounds, if it produced a confidence percentage of 100% for tickle laughter, 50% for tickle-miss, 25% for tickle-chase, this could potentially indicate the evolution of laughter from highlighting an individual’s own incongruity to the individual highlighting another’s. Deep learning could also be employed in analysing laughter vocalizations in both humans and non-human apes, whereby a convolutional neural network could be trained on a human laughter then applied to chimpanzee vocalizations, where confidences of types of laughter could help identify similar signals, traits, and evolutionary pathways.

It has been shown that with enough data, deep-learning neural networks can learn physical properties such as the chemical space in molecular design [55] and have been used to predict the temporal evolution of fluid flow within neural network latent spaces [56]. Latent spaces have been used in style-based neural networks to generate realistic images of a variety of animals and objects. The original StyleGAN (style-based generator architecture for generative adversarial networks) [57] can generate synthetic photorealistic images, and its architecture allows for an unsupervised separation of attributes and stochastic variation in generated images, which enables realistic interpolation from an image of one face to another. As such, deep learning using latent space exploration could be used to identify evolutionary pathways via the neural network’s ability to organize data in latent space, in a similar way to that of pollen images as described by [58]. For example, a StyleGAN type neural network trained on of different types of laughter spectral images, such as joyous, taunting and tickling, from a variety of individuals and even species, could enable understanding of evolutionary pathways of laughter via exploration from laughter type to another laughter type, or from one species to another species for the same laughter type. Further to this, different latent space vectors could be determined, such as certain frequencies that play a key role throughout different spectra, to help identify pathways of laughter’s vocal development.

However, such neural networks require a large amount of data, with the original StyleGAN neural network utilizing 70,000 high-quality images (https://github.com/NVlabs/ffhq-dataset) and the pollen morphology paper using nearly 3000 high-quality images. Therefore, to be able to create a large enough number of acoustic spectra for training a neural network for realistic synthetic acoustic spectra, it will likely be necessary to employ a convolutional neural network to be able to identify certain types of laughter from many hours of videos of laughter, be it human or chimpanzee vocalizations. In addition, the same neural network will be needed to identify synthetic acoustic spectra generated by the style-based neural network to allow for interpolation of acoustic spectra in latent space between confidently known types of laughter, such as tickling induced laughter and joyous laughter. However, whilst datasets of human faces, animals and wildlife videos are readily available, to be able to acquire the correct large amount of data specifically of laughter types from different species would require a significant undertaking and most likely collaboration between different research groups and study areas. One way to achieve this would be to create a global dataset of every laughter type with corresponding videos and spectra using contributions by groups and institutes from around the world.

## Data Availability

Data sharing is not applicable to this article as no new data were created or analyzed in this study.
